# A Novel Direction-of-Arrival Estimation via Phase Retrieval with Unknown Sensor Gain-and-Phase Errors

**DOI:** 10.3390/s19122701

**Published:** 2019-06-15

**Authors:** Lingwen Zhang, Siliang Wu, Ao Guo, Wenkao Yang

**Affiliations:** School of Electronic and Information Engineering, Beijing Jiaotong University, Beijing 100044, China; zhanglw@bjtu.edu.cn (L.Z.); 18125011@bjtu.edu.cn (A.G.); yangwk@bjtu.edu.cn (W.Y.)

**Keywords:** direction-of-arrival (DOA) estimation, gain-and-phase errors, phase retrieval

## Abstract

In signal array processing, high-resolution direction-of-arrival (DOA) estimation algorithms work well on the assumption that the system models are perfect. However, in practicality, there are imperfect system models in which sensor gain-and-phase errors are considered. In this paper, we propose a novel framework that can effectively solve direction-of-arrival estimation tasks in the presence of sensor gain-and-phase errors. In contrast to existing approaches based on phase retrieval, our method eliminates gain errors by using the compensated covariance matrix. Meanwhile, we propose a data preprocessing method by taking only one column of the compensated covariance matrix without losing any magnitude information. Additionally, the phase retrieval problem is formed by the proposed data preprocessing method. Furthermore, the phase retrieval problem is solved by the recently proposed sparse feasible point pursuit algorithm, and DOA estimates are obtained. To prevent the model from ambiguities, we employ the known DOA to place reference sources. Numerical results show that the proposed scheme achieves better performance compared to state-of-the-art approaches.

## 1. Introduction

Direction-of-arrival (DOA) estimation of plane waves using sensor arrays is a key problem which is frequently encountered in many applications, such as radar, sonar, biomedical engineering, and astronomy. Various high-resolution algorithms for DOA estimation, such as Multiple Signals Classification (MUSIC) [1], Estimation of Signal Parameters via Rotational Invariance Technique (ESPRIT) [2], and the Propagator method [3] have been proposed. However, this class of approaches is based on the assumption of the ideal array manifold. In practical applications, there always exist some array perturbations, such as sensor gain-and-phase errors, which will cause severe degradation in performance [4,5,6].

### 1.1. Related Work

Over the last four decades, a number of approaches have been proposed to calibrate the gain-and-phase errors. The approaches developed in [7,8] have excellent performance; however, these approaches require a multidimensional search so that they are complicated and time-consuming, and they require calibration signals with known directions. Paulraj and Kailath [9] first investigated the problem of DOA estimation for uniform linear arrays (ULA) in the presence of unknown gain-and-phase errors, and proposed an approach to correct the array perturbations by estimating these errors, but could not provide a unique solution.

The approaches in [10,11] can simultaneously estimate the DOAs of signals and array parameters. Unfortunately, these approaches suffer from suboptimal convergence because of the joint iteration between DOA estimation and array parameter estimation, and they are based on the assumption that the array errors are small.

Wylie et al. [12] also considered the problem in the presence of phase errors, and developed an approach that can obtain a unique solution with some heuristic constraints of phase errors. The primary contribution of this work is a simple, robust (and hence, practically useful) linear least-square (LS) -based algorithm for self-calibration of linear-equispaced (LES) arrays with constant but unknown phase errors that do not require any a priori knowledge of the DOA of any of the received signals, and is consequently useful in situations where a cooperating source may not exist, or is temporarily unavailable. LS estimation of the unknown DOA and array phase response is accomplished via the imposition of a novel constraint, and the resultant estimator bias is shown to be inversely proportional to the number of array elements.

In [13], Li and Er modified the tedious approach in [9] and proposed a class of simplified calibration algorithms based on different diagonal lines of the covariance matrix. They concluded that more diagonals may not result in better performance. Moreover, they presented the theoretical performance of the gain-and-phase-errors estimation. The simplified approach in [13] is superior to the original in [9]. A novel gain-and-phase calibration approach using independent component analysis was proposed in [14], and able to achieve better performance than conventional approaches, but only applicable to no-Gaussian sources.

Zhao et al. [15] proposed a sparse-based DOA estimation approach under sensor gain-and-phase uncertainties, and the approach utilized the sparsity of DOAs and uncertainty matrix and designed an optimization problem for a joint estimation method. Then, an iterative two-step process was proposed to solve the optimization problem, but Zhao’s approach is based on the assumption that the array errors are small.

However, these calibration approaches are sensitive to phase errors, and the DOA estimation level drops as the phase errors increase [7,8,9,10,11,12,13,14,15]. This serves as motivation for the introduction of a DOA estimation approach that is robust against phase errors.

The approach in [16,17] can perform independently of the phase errors, but are limited to the configuration of the arrays. These approaches cannot be applied to linear arrays. Moreover, they are not applicable to cases where only one signal is observed.

### 1.2. Motivation and Contribution

Recently, the study on phase retrieval has attracted attention in signal processing [18,19,20]. The problem of phase retrieval refers to recovering a signal from its magnitude measurements, and appears in various fields, such as electron microscopy [21,22], crystallography [23], and optical imaging [24,25], where it is difficult to measure the phase information [26]. To remove the effects of phase errors, Kim et al. first applied the theory of phase retrieval in DOA estimation and proposed a new DOA estimation approach from magnitude-only measurements [27]. Kim’s approach does not make use of sensor phase information, so it is robust to phase errors. However, the gain errors were not eliminated in the approach; hence, the DOA estimating performance of Kim’s approach becomes degraded as the gain errors increase.

In this paper, we develop a direction-of-arrival (DOA) estimation approach for uniform linear array (ULA) in the presence of gain-and-phase errors based on phase retrieval. The proposed approach can solve the potential impracticality of Kim’s approach when gain-and-phase errors coexist. Firstly, we start off by estimating the gain errors, and propose a data preprocessing method for the compensated covariance matrix by taking one column of the matrix, though we include all the magnitude information. In addition, the phase retrieval problem is formed by the proposed data preprocessing method. The estimates of ambiguities are gotten rid of by introducing reference sources. By combining this with the phase retrieval technique in [28], the proposed DOA estimation approach outperforms Kim’s approach in the presence of gain-and-phase errors.

### 1.3. Organizations

The remainder of this paper is arranged as follows. In Section 2, we present the direction-of-arrival estimation system model in the presence of gain-and-phase errors. In Section 3, we propose a new DOA estimation framework based on phase retrieval. Section 4 shows the simulation results of DOA estimation performance for the proposed approach, and conclusions are given in Section 5.

## 2. Data Model

Consider a ULA consisting of *M* sensors with element spacing *d*. Let λ denote the wavelength of the carrier signal. Assume there are *Q* far-field narrowband source signals impinging on the ULA. The arriving direction of the *q*th signal is specified by $\theta_{q},q=1,2,\ldots ,Q$. Then, the steering vector of the ULA is

(1) $$a\left(\theta_{q}\right)={\left[1,e^{j2\pi d\sin\theta_{q}/\lambda },\cdots ,e^{j2\pi \left(M-1\right)d\sin\theta_{q}/\lambda }\right]}^{T}.$$

The signal received by the ULA array is given by the M×1 vector

(2) x(t)=As(t)+n(t),

where $A=[a\left(\theta_{1}\right),a\left(\theta_{2}\right),\cdots ,a\left(\theta_{Q}\right)]$ is the M×Q steering matrix of the ULA with respect to the directions of the incoming source signals of *Q*, s(t) is the Q×1 source vector representing *Q* source waveforms arriving at the ULA, and n(t) is the M×1 additive Gaussian noise vector.

In this paper, the superscripts T and H represent the transpose and conjugate transpose operations, respectively. For the (2) model, three assumptions are introduced, which are considered to hold throughout the paper.

Assumption1: The sources are zero-mean, stationary, and unrelated.

Assumption2: The source s(t) is independent of the additive noise n(t).

Assumption3: n(t) is assumed to be stationary, zero-mean, and spatially white Gaussian.

Denote the gain-and-phase error of the *m*th sensor with $\rho_{m}$ and $\varphi_{m}$, respectively. The received signal in the presence of gain-and-phase errors is

(3) x(t)=GΦAs(t)+n(t),

where Φ and G are diagonal matrices, and their *m*th diagonal elements are $e^{j\varphi_{m}}$ and $\rho_{m}$, respectively. Without loss of generality, we assume that $\rho_{1}=1$.

## 3. DOA Estimation Framework Based on Phase Retrieval

### 3.1. Gain Errors Estimation from Covariance Matrix

From (3), the covariance matrix $R=E[x\left(t\right)x^{H}\left(t\right)]$ can be expressed as

(4) $$R=G\Phi AR_{s}A^{H}\Phi^{H}G^{H}+\sigma_{n}^{2}I_{M},$$

where $R_{s}=E\left[s\left(t\right)s{\left(t\right)}^{H}\right]=\mathrm{diag}\left(\left[\sigma_{1}^{2},\ldots ,\sigma_{Q}^{2}\right]\right)$, $\sigma_{q}^{2}$ denotes the power of the *q*th source, $\sigma_{n}^{2}$ denotes the power of the noise, and $I_{M}$ is an M×M identity matrix.

The eigendecomposition of R is given as

(5) $$R=\sum_{m=1}^{M}v_{m}u_{m}u_{m}^{H},$$

where the eigenvalues ${\left\{v_{m}\right\}}_{m=1}^{M}$ are arranged in descending order and $u_{m}$ is the corresponding eigenvector. Then, $\sigma_{n}^{2}$ can be estimated as

(6) $${\hat{\sigma }}_{n}^{2}=\frac{1}{M-Q}\sum_{m=Q+1}^{M}v_{m}.$$

Define the *m*th diagonal element R of R(m,m). From (4), we have

(7) $$R\left(m,m\right)=\rho_{m}^{2}\sum_{q=1}^{Q}\sigma_{q}^{2}+\sigma_{n}^{2}.$$

Recall that $\rho_{1}=1$, where the gain errors can be estimated as

(8) $${\hat{\rho }}_{m}=\sqrt{\frac{R\left(m,m\right)-{\hat{\sigma }}_{n}^{2}}{R\left(1,1\right)-{\hat{\sigma }}_{n}^{2}}},m=2,3,\ldots ,M.$$

### 3.2. Gain Errors Calibration

As the gain errors and noise power have been estimated, we are going to find a way to calibrate the gain errors. Since the information on arriving angle is included in the covariance matrix, it can be a feasible way to estimate the arriving angles from the covariance matrix, meaning that we need to eliminate the gain errors in this matrix.

As the noise power is estimated from (6), we can remove the noise in the covariance matrix first:

(9) $$R-{\hat{\sigma }}_{n}^{2}I_{M}\approx G\Phi AR_{s}A^{H}\Phi^{H}G^{H}$$

Using the estimated gain errors from (8), we can eliminate the gain errors by compensating for the covariance matrix:

(10) $$R_{c}={\hat{G}}^{-1}\left(R-{\hat{\sigma }}_{n}^{2}I_{M}\right){\left({\hat{G}}^{-1}\right)}^{H}\approx \Phi AR_{s}A^{H}\Phi^{H}.$$

where $\hat{G}=\mathrm{diag}\left(1,{\hat{\rho }}_{2},\ldots ,{\hat{\rho }}_{M}\right)$. For the compensated covariance matrix $R_{c}$, we can find that the impact of gain errors and noise can be removed if they are correctly estimated. The next object is to eliminate the phase errors in the compensated covariance matrix, $R_{c}$.

### 3.3. A Simple Data Preprocessing Method

For the compensated covariance matrix $R_{c}$, we propose a new simple data preprocessing method in order to simplify the complexity of the problem. Define the mnth element of $R_{c}$ as $r_{mn}$; $r_{mn}$ can then be expressed as (assume d=λ/2)

(11) $$r_{mn}=e^{j(\varphi_{m}-\varphi_{n})}\sum_{q=1}^{Q}\sigma_{q}^{2}e^{j(\pi \left(m-n\right)\sin\theta_{q})}.$$

Let $B=\sum_{q=1}^{Q}\sigma_{q}^{2}e^{j(\pi \left(m-n\right)\sin\theta_{q})}$, and Equation (11) can then be written as

(12) $$r_{mn}=e^{j(\varphi_{m}-\varphi_{n})}B.$$

Based on the Euler Theorem, $r_{mn}=B\left(\cos\left(\varphi_{m}-\varphi_{n}\right)+j\sin\left(\varphi_{m}-\varphi_{n}\right)\right)$. Therefore, taking the magnitude squared of $r_{mn}$, we obtain

(13) $${\left|r_{mn}\right|}^{2}={\left|B\right|}^{2}={\left|\sum_{q=1}^{Q}\sigma_{q}^{2}e^{j(\pi \left(m-n\right)\sin\theta_{q})}\right|}^{2}.$$

It is easy to see that the magnitude information of $r_{mn}$ is independent of phase errors, but depends on the phase information related to the arriving angle $\theta_{q}$. This motivates the introduction of a DOA estimation approach based on the magnitude information of $r_{mn}$. Taking the first column of $R_{c}$, we obtain an M×1 vector

(14) $$r=\left[\sum_{q=1}^{Q}\sigma_{q}^{2},e^{j(\varphi_{2}-\varphi_{1})}\sum_{q=1}^{Q}\sigma_{q}^{2}e^{j(\pi \sin\theta_{q})},\ldots ,\right.{\left.e^{j(\varphi_{M}-\varphi_{1})}\sum_{q=1}^{Q}\sigma_{q}^{2}e^{j(\pi \left(M-1\right)\sin\theta_{q})}\right]}^{T}.$$

Then, taking the magnitude squared of r, we obtain

(15) $$y=\left[{\left|\sum_{q=1}^{Q}\sigma_{q}^{2}\right|}^{2},{\left|\sum_{q=1}^{Q}\sigma_{q}^{2}e^{j(\pi \sin\theta_{q})}\right|}^{2},\ldots ,\right.{\left.{\left|\sum_{q=1}^{Q}\sigma_{q}^{2}e^{j(\pi \left(M-1\right)\sin\theta_{q})}\right|}^{2}\right]}^{T},$$

where $y={\left|r\right|}^{2}$ and the absolute value operator is applied element-wise to the vector r. The magnitude squared of the *n*th column of $R_{c}$ can be expressed as

(16) $$y_{n}=\left[{\left|\sum_{q=1}^{Q}\sigma_{q}^{2}e^{j(\pi \left(1-n\right)\sin\theta_{q})}\right|}^{2},\right.{\left|\sum_{q=1}^{Q}\sigma_{q}^{2}e^{j(\pi \left(2-n\right)\sin\theta_{q})}\right|}^{2},\ldots ,{\left.{\left|\sum_{q=1}^{Q}\sigma_{q}^{2}e^{j(\pi \left(M-n\right)\sin\theta_{q})}\right|}^{2}\right]}^{T},$$

where $y_{n}={\left|r_{n}\right|}^{2}$ and $r_{n}$ denotes the *n*th column of $R_{c}$.

Based on the following equation:

(17) $${\left|\sum_{q=1}^{Q}\sigma_{q}^{2}e^{j(\pi \left(m-n\right)\sin\theta_{q})}\right|}^{2}={\left|\sum_{q=1}^{Q}\sigma_{q}^{2}e^{j(\pi \left(n-m\right)\sin\theta_{q})}\right|}^{2},$$

we have Lemma 1 about the magnitude squared of the elements in the compensated covariance matrix $R_{c}$.

**Lemma** **1.** *For any element in $y_{n},n=2\ldots M$, there must be an equivalent element in y. Therefore, we can say that y contains all the magnitude information of $R_{c}$.*

**Proof.**  The elements in y are given as $\left|\sum_{q=1}^{Q}\sigma_{q}^{2}e^{j(\pi \left(m-1\right)\sin\theta_{q})}\right|,m=1\ldots M$, and the elements in $y_{n}$ are expressed as $\left|\sum_{q=1}^{Q}\sigma_{q}^{2}e^{j(\pi \left(m-n\right)\sin\theta_{q})}\right|,m=1\ldots M$. Because n=2…M, the lemma can be proved by (16). □

Note that y contains all the magnitude information of $R_{c}$, and we can use $y={\left|r\right|}^{2}$, only a column of $R_{c}$, to form the phase retrieval problem in order to reduce the computational cost. Therefore, we only use one column of $R_{c}$ without missing the magnitude information. The simple data preprocessing method for the compensated covariance matrix helps us to reduce the redundancy of the matrix and form the phase retrieval problem easily.

### 3.4. A New Way to Eliminate the Phase Errors

r can be written as

(18) $$r=\Phi^{\prime }\mathrm{Ap},$$

where $p={\left[\sigma_{1}^{2},\sigma_{2}^{2},\ldots ,\sigma_{Q}^{2}\right]}^{T}$, and $\Phi^{\prime }$ can be regarded as a new phase error matrix: $\Phi^{\prime }=\mathrm{diag}\left(\left[1,e^{j(\varphi_{2}-\varphi_{1})},\ldots ,e^{j(\varphi_{M}-\varphi_{1})}\right]\right)$. In order to convert the DOA estimating problem into a nonlinear optimization in a sparse framework, we assume the source’s possible DOAs comply with a grid of *S* points, with S≫Q. Consider the estimation error for the gain errors and noise power, where (17) can be revised as

(19) $$r\approx \Phi^{\prime }\tilde{A}\tilde{p},$$

where $\tilde{p}$ is defined as an S×1 sparse vector that only has *Q* non-zero elements representing source powers, and $\tilde{A}$ is defined as a M×S dictionary matrix with a *s*th column:

(20) $$a\left(\theta_{s}\right)={\left[1,e^{j2\pi d\sin\theta_{s}/\lambda },\cdots ,e^{j2\pi \left(M-1\right)d\sin\theta_{s}/\lambda }\right]}^{T},$$

where $\theta_{s}$ refers to a possible DOA. Our goal is to determine the *Q* non-zero elements of $\tilde{p}$ from the knowledge of r and $\tilde{A}$. The support of the sparse vector $\tilde{p}$ has a one-to-one relation with the *Q* arriving angles $\theta_{q},q=1,2,\ldots ,Q$. Based on ${\left|\Phi^{\prime }\tilde{A}\tilde{p}\right|}^{2}={\left|\tilde{A}\tilde{p}\right|}^{2}$, we can obtain

(21) $$y={\left|r\right|}^{2}\approx {\left|\tilde{A}\tilde{p}\right|}^{2}.$$

Therefore, we have converted the DOA estimation problem into a phase retrieval problem [13]. The rows of $\tilde{A}$ can be regarded as the known measurement vectors. We can now propose estimating the support of $\tilde{p}$ from $\tilde{A}$ and y, and we can find that the phase errors are totally removed in (21) by taking the magnitude squared of the elements in r.

### 3.5. Phase Ambiguities Solution

However, the phase ambiguity problem associated with phase retrieval solutions should be considered in the DOA estimation. It is significant to resolve the phase ambiguity problem to get a unique solution.

Consider the scenario of two sources, assume the compensated covariance matrix is approximated correctly, and let $\beta_{q}=\pi \left(m-1\right)\sin\theta_{q}$, where $y_{m}$ can be written as:

(22) $$\begin{array}{c} y_{m}={\left|\sum_{q=1}^{2}\sigma_{q}^{2}e^{j\beta_{q}}\right|}^{2}={\left|\sigma_{1}^{2}e^{j\beta_{1}}+\sigma_{2}^{2}e^{j\beta_{2}}\right|}^{2}\\ =\sigma_{1}^{4}+\sigma_{2}^{4}+2\sigma_{1}^{2}\sigma_{1}^{2}\cos\left(\beta_{1}-\beta_{2}\right). \end{array}$$

Assume the source power is known, and the arriving angle can be estimated from Equation (22). To simplify the phase ambiguity problem, let $\sigma_{1}^{2}=\sigma_{2}^{2}=1/\sqrt{2}$, $\Delta \beta =\beta_{1}-\beta_{2}$. Equation (22) then becomes

(23) $$y_{m}=1+\cos\Delta \beta .$$

**Figure 1:**
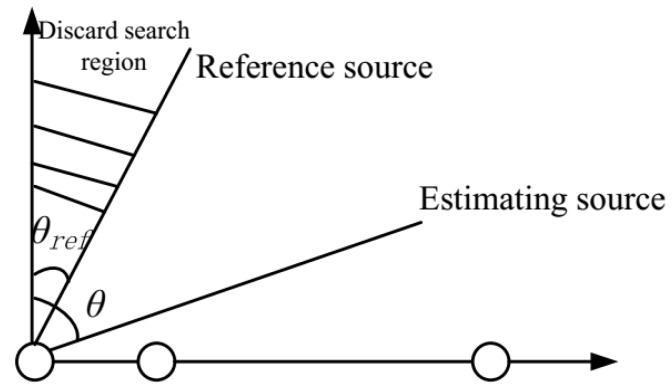
Using a reference source to solve the phase ambiguities.

The solution Δβ to (23) is subject to phase ambiguities, which consist of phase shift ambiguity and phase mirroring ambiguity. The phase shift ambiguity refers to the case that there exists an arbitrary $\beta_{i}$, $\Delta \beta =\beta_{1}-\beta_{2}=\left(\beta_{1}+\beta_{i}\right)-\left(\beta_{2}+\beta_{i}\right)$. The phase mirroring ambiguity originates from the fact that Δβ and −Δβ can be the two solutions to (23). The phase ambiguity can be removed by placing known reference sources, where the DOA of a source can be estimated unambiguously if the source is restricted to only one side of the reference source. For example, if the DOA of the reference source is set at $\theta_{ref}=0$ rad, the estimating DOA can be estimated unambiguously if it is restricted to the region [0,π/2]. As depicted in Figure 1, the angle ambiguities problem can be solved after a reference target is placed at the cost of a small loss in the visible region. For the multiple-sources DOA estimation problem, the ambiguity can be removed by setting multiple reference sources.

### 3.6. DOA Estimation Using Sparse Least-Square Feasible Point Pursuit (LS-FFP) Approach

Based on (21), the DOA estimation problem can be considered as the optimization problem

(24) $$\min_{\tilde{p}}{∥y-{\left|\tilde{A}\tilde{p}\right|}^{2}∥}_{2}^{2}.$$

Let $b_{m}$ denote the *m*th row of $\tilde{A}$, where (24) can be recast as

(25) $$\min_{\tilde{p}}\sum_{m=1}^{M}{\left(y_{m}-{\tilde{p}}^{H}B_{m}\tilde{p}\right)}^{2},$$

where $B_{m}={b_{m}}^{H}b_{m}$ and (22) can be recognized as the least-squares (LS) formulation for phase retrieval. To solve (25), we use the sparse Least-Square Feasible Point Pursuit (LS-FFP) algorithm, a phase retrieval algorithm that exploits sparsity [28]. Firstly, (25) is recast in the following equivalent form:

(26) $$\begin{array}{c} \min_{w,\tilde{p}}\;{∥w∥}_{2}^{2}\\ s.t.\;\;\;{\tilde{p}}^{H}B_{m}\tilde{p}+w_{m}=y_{m},\forall m, \end{array}$$

where $w={\left[w_{1},\ldots ,w_{M}\right]}^{T}$, and the equality constraints in (26) are rewritten as

(27) $${\tilde{p}}^{H}B_{m}\tilde{p}+w_{m}\leq y_{m},$$

(28) $${\tilde{p}}^{H}B_{m}\tilde{p}+w_{m}\geq y_{m}.$$

It is clear that (28) is a non-convex constraint. In order to transform the optimization problem into a convex one, following the principles in [29], (28) can be replaced by

(29) 2Re{zHBmp˜}+wm+hm≥ym+zHBmz,

where z is an S×1 random vector, and $h_{m}\geq 0$ is a slack variable. We thus obtained the following convex, quadratically constrained quadratic programming (QCQP) by:

(30) minp˜,w,hw22+λ∑i=1Mhms.t.2Re(zHBmp˜)+wm+hm≥zHBmz+ymp˜HBmp˜+wm≤ymhm≥0,∀m,

where $h={\left[h_{1},\ldots h_{M}\right]}^{T}$, and λ trades off the original objective function and the slack penalty term. For sparse LS-FFP, we have:

(31) minp˜,w,hw22+λ∑i=1Mhm+μp˜1s.t.2Re(zHBmp˜)+wm+hm≥zHBmz+ymp˜HBmp˜+wm≤ymhm≥0,∀m,

where $l_{1}$-norm ${∥\tilde{p}∥}_{1}$ is an ideal description of sparsity, and μ balances the objective function and the sparsity of $\tilde{p}$.

As indicated in Section 3.5, reference sources should be added to resolve the ambiguity problem. In this paper, the sources are random variables distributed according to the standard Gaussian distribution, so the average power of each source is 1. For the scenario of the single-source DOA estimation, a reference source is set at $\theta_{r}=0$, and the estimating source is coming from $\left[0,\pi /2\right]$ rad. The M×S dictionary matrix is $\tilde{A}=\left[a\left(0\right),a\left(\Delta \right),\cdots ,a\left(\pi /2-\Delta \right),a\left(\pi /2\right)\right]$, and S=π/2Δ+1. Thus, we modified (31) to the following optimization problem by adding two constraints,

(32) minp˜,w,hw22+λ∑i=1Mhm+μp˜1s.t.2Re(zHBmp˜)+wm+hm≥zHBmz+ymp˜HBmp˜+wm≤ymhm≥0,∀mps≥0,∀sp1=1,

where $p_{s}$ denotes the power of the *s*th source, and $p_{1}=1$ describes the reference source with direction-of-arrival (DOA) $\theta_{r}\;=0$ rad. Using the convex optimization toolbox convex (CVX) [30], we were able to solve the convex quadratically constrained quadratic programming (QCQP) (32) in MATLAB.

LS-FPP is an iterative algorithm starting with a random initial S×1 vector z. In the *k*th iteration, we solve (32) to obtain ${\tilde{p}}_{k}$, then setting $z_{k+1}={\tilde{p}}_{k}$. Since the optimal value of the cost function in each iteration step is non-increasing [28], the iteration stops until the difference of the optimal value between two adjacent iterations is less than a given threshold. As $\tilde{p}$ is estimated, the DOA estimates can be obtained by seeking the peaks of $\tilde{p}$.

Consequently, the proposed approach for DOA estimation is summarized as follows:

Step 1: Estimate the covariance matrix with L snapshots

(33) $$\hat{R}=\frac{1}{L}\sum_{l=1}^{L}x\left(t_{l}\right)x^{H}\left(t_{l}\right).$$

Step 2: Estimate the noise power and gain errors by (6) and (8).

Step 3: Calculate the compensated covariance matrix $R_{c}$ as (10).

Step 4: Take the first column of $R_{c}$ and construct the data model as (18).

Step 5: Use the sparse LS-FPP approach to solve the convex QCQP (32) and obtain $\tilde{p}$.

Step 6: Obtain DOA estimates by seeking the peaks of $\tilde{p}$.

### 3.7. Computational Complexity

Computational complexity of the proposed DOA estimation framework lies in three parts: gain errors estimation, compensated covariance matrix construction, and DOA estimation using the Sparse LS-FFP approach. For the gain errors estimation part, the computational complexity is $O(M^{2}L+M^{3})$, which comes from the covariance matrix calculation and eigenvalue decomposition of the covariance matrix. For the compensated covariance matrix construction part, the computational complexity is $O(M^{3})$, which comes from Equation (10). For the part where the DOA estimation was done using the Sparse LS-FFP approach, the computational complexity mainly comes from solving the optimization problem (32), which is $O({\left(S+3M\right)}^{3.5})$. Assuming the maximum iteration is $I_{\max}$, the complexity of the part where DOA estimation is done using the Sparse LS-FFP approach is $O(I_{\max}{\left(S+3M\right)}^{3.5})$.

**Figure 2:**
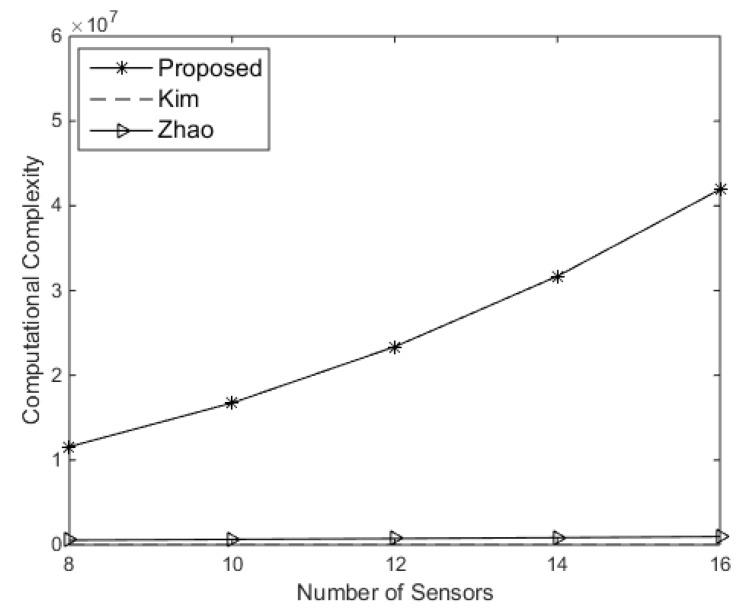
Complexity comparison of different approaches versus the number of sensors.

The computational complexity of Kim’s approach is $O(I_{\max}{\left(S+M\right)}^{2})$. For the approach in [15], it cost $O(I_{\max}{\left(S+M\right)}^{3})$. Figure 2 shows the computational complexity comparison of the proposed approach and other existing approaches. The maximum iteration is assumed to be $I_{\max}=10$ and S=30. It can be seen that the proposed approach requires more computational complexity to improve performance.

## 4. Numerical Simulations

In this section, numerical simulations are run to demonstrate the performance of the proposed DOA estimation approach. All the simulations are based on a ULA with M=32 sensors, and the sensor spacing d=0.5λ. The number of snapshot is L=100. Let Δ=π/60 rad, so a grid of S=31 points is uniformly spaced over the $\left[0,\pi /2\right]$ rad. A reference source of the known DOA is set at $\theta_{ref}=0$ rad, and the estimating source is located at θ=2π/15 rad. We also evaluated the performance of the proposed approach for the multiple sources estimation, where two known reference sources were set at $\theta_{ref1}=0$ rad and $\theta_{ref2}=\pi /15$ rad, and the estimating sources are located at $\theta_{1}=2\pi /15$ rad and $\theta_{2}=\pi /5$ rad. The input signal-to-noise ratio (SNR) of the *q*th signal is defined as $\mathrm{SNR}=-10\log_{10}\sigma_{n}^{2}$. The gain-and-phase errors were generated by the following formulas, respectively:

(34) $$\rho_{m}=1+\sqrt{12}\sigma_{\rho }\beta_{m},$$

(35) $$\varphi_{m}=\sqrt{12}\sigma_{\varphi }\eta_{m},$$

where $\beta_{m}$ and $\eta_{m}$ are uniform random variables in interval $\left[-0.5,0.5\right]$, $\sigma_{\rho }$, and $\sigma_{\varphi }$ are the standard deviations of gain-and-phase errors, respectively. To compare with Kim’s approach, we used the probability of error to evaluate the performance. The probability of error is defined as the ratio of incorrectly estimated DOAs to 100 simulations. Moreover, we compare this with a state-of-the-art sparse DOA estimation approach proposed by Zhao et al. [15].

**Figure 3:**
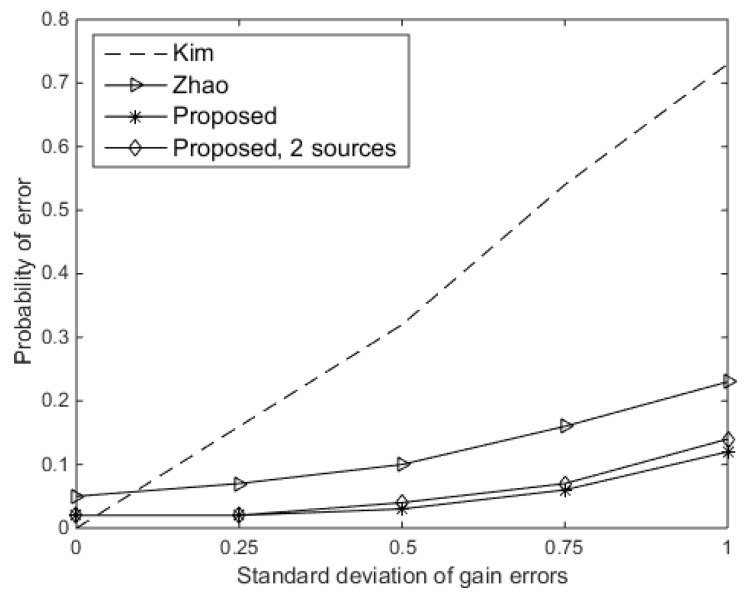
Probability of error versus the standard deviation of gain errors.

Firstly, we compared the probability of error of the DOA estimation versus the standard deviation of gain errors ($\sigma_{\rho }$) for the existing approaches and the proposed approach of one or two sources. Here, the SNR was set at 10 dB, and the standard deviation of phase errors ($\sigma_{\varphi }$) was assumed to be 0.3π rad. As shown in Figure 3, the performance of Kim’s approach noticeably began to deteriorate as $\sigma_{\rho }$ increased. For the proposed approach, it has a probability of error lower than 0.1 for $\sigma_{\rho }\leq 0.75$. Therefore, the proposed approach performs more robustly than Kim’s approach for a wide range of $\sigma_{\rho }$. The proposed approach performs slightly better than Zhao’s approach; however, Kim’s approach is better when the gain errors do not exist. In the next two experiments, the standard deviation of gain errors is set at 0.5, and Kim’s approach is not considered in the next experiments because it is not suitable when the gain errors exist, as we can know from Figure 3.

**Figure 4:**
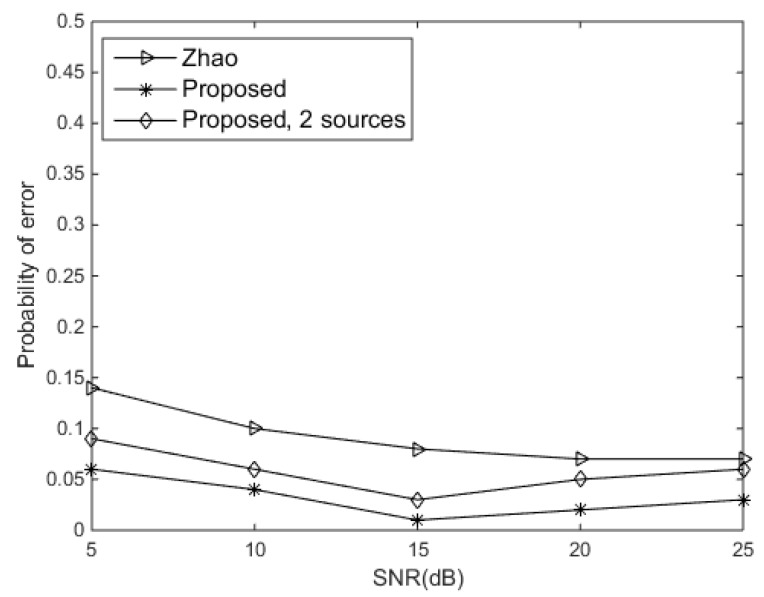
Probability of error versus signal-to-noise ratio (SNR).

Then, we evaluated the probability of error of the DOA estimation versus SNR for the proposed approach of one or two sources. The standard deviation of phase errors ($\sigma_{\varphi }$) was assumed to be 0.3π rad, and the standard deviation of gain errors ($\sigma_{\rho }$) was set at 0.5. From Figure 4, it was shown that the probability of error of the proposed approach is lower than 0.1 for all SNRs. The proposed approach was shown to provide better performance than Zhao’s approach. It is remarkable that the proposed approach is robust to noise.

**Figure 5:**
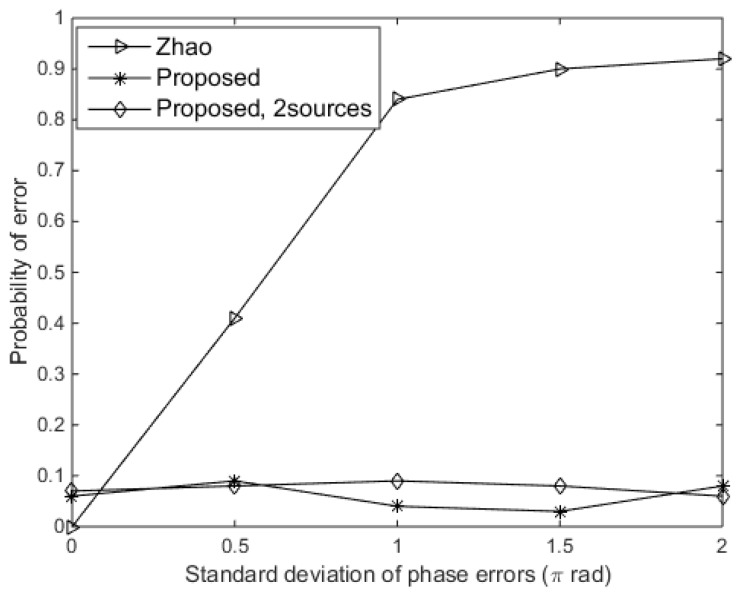
Probability of error versus the standard deviation of phase errors.

To demonstrate the effect of phase errors on DOA estimation, we evaluated the probability of error of the DOA estimation for the proposed approach with respect to the standard deviation of phase errors from 0 rad to 2π rad. Here, the standard deviation of gain errors ($\sigma_{\rho }$) was set at 0.5, and the SNR was 10 dB. Figure 5 shows that the probability of error of the proposed approach is lower than 0.1 as the standard deviation of phase errors varies. However, the performance of Zhao’s approach becomes degraded when $\sigma_{\varphi }>0.3\pi$ rad. Zhao’s approach was based on the assumption that the array errors are small [15], and it is indicated that the phase errors have no effect on the proposed approach, which is a key benefit based on phase retrieval.

**Figure 6:**
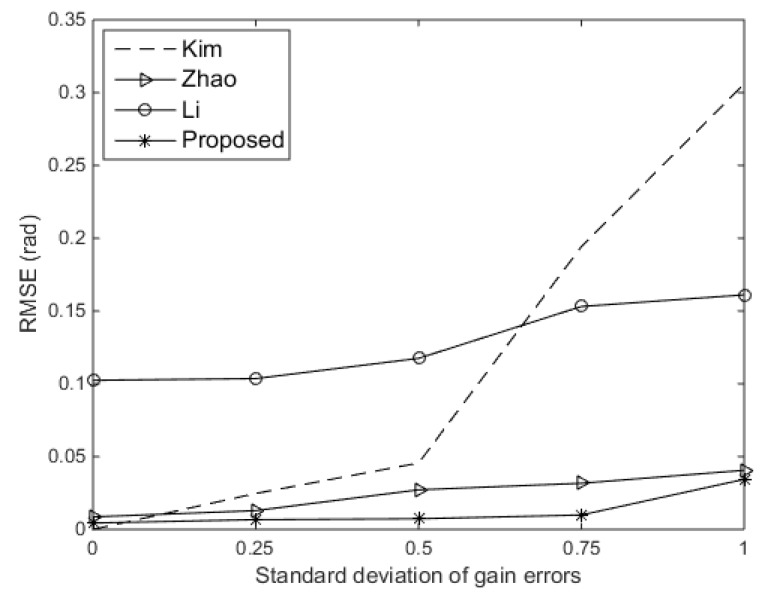
Root mean square error (RMSE) versus the standard deviation of gain errors.

**Figure 7:**
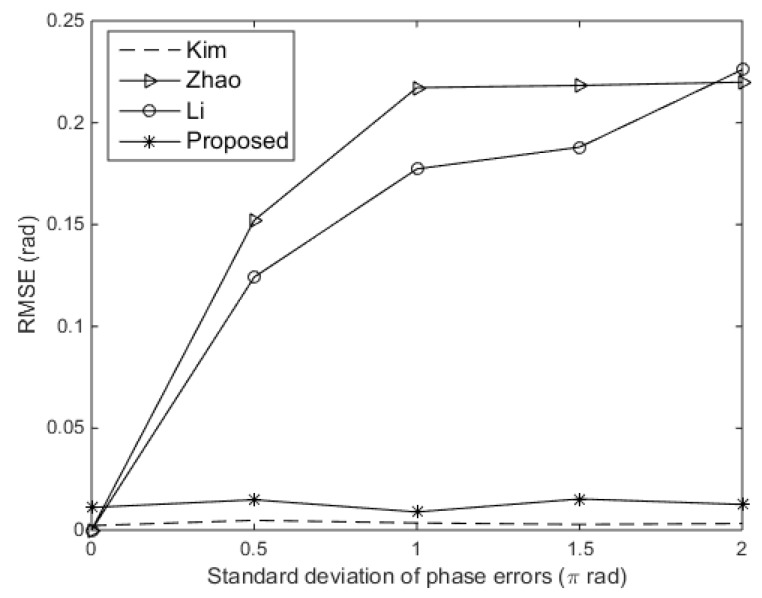
RMSE versus the standard deviation of phase errors.

In order to explore the results better, we also evaluated the root mean square error (RMSE) on the DOA estimates. We compared the RMSE of the DOA estimates versus the standard deviation of gain errors for the existing approaches and the proposed approach. The SNR was set at 10 dB, and the standard deviation of phase errors was assumed to be 0.3π rad. We also compared with another existing techniques in [13]. As shown in Figure 6, the proposed approach performs better than other existing approaches. Then, we compared the RMSE of the DOA estimates versus the standard deviation of gain errors for the existing approaches and the proposed approach. The SNR was set at 10 dB, and the standard deviation of gain errors was set at 0.15. Shown in Figure 7, we can see that the proposed approach and Kim’s approach perform independently of phase errors. Kim’s approach even performs slightly better than the proposed approach, because the standard deviation of gain errors is relatively small in this experiment. However, Zhao’s approach and Li’s approach perform badly when the standard deviation of phase errors rise to 0.5π rad.

## 5. Conclusions

In this paper, we presented a new DOA estimation approach for ULA in the presence of gain-and-phase errors based on phase retrieval. In contrast to the existing DOA estimation approach using phase retrieval, the proposed approach can provide more robust DOA-estimating performance as the gain errors increase. Meanwhile, because the effect of phase errors is eliminated by taking the magnitude squared of the elements in the compensated covariance matrix, the proposed approach performs independently of phase errors.

## References

[1] Schmidt R.O. (1986). Multiple Emitter Location and Signal Parameter Estimation. IEEE Trans. Antennas Propag..

[2] Roy R., Kailath T. (1989). ESPRIT-estimation of signal parameters via rotational invariance techniques. IEEE Trans. Acoust. Speech Signal Process..

[3] Marcos S., Marsal A., Benidir M. (1995). The propagator method for source bearing estimation. Signal Process..

[4] Godara L. (1985). The effect of phase-shifter errors on the performance of an antenna-array beamformer. IEEE J. Ocean. Eng..

[5] Stoica P., Wang Z., Li J. (2005). Extended derivations of MUSIC in the presence of steering vector errors. IEEE Trans. Signal Process..

[6] Ferréol A., Larzabal P., Viberg M. (2010). Statistical analysis of the MUSIC algorithm in the presence of modeling errors, taking into account the resolution probability. IEEE Trans. Signal Process..

[7] Ng B.C., See C.M.S. (1996). Sensor-array calibration using a maximum-likelihood approach. IEEE Trans. Antennas Propag..

[8] See C.M.S. (1995). Method for array calibration in high-resolution sensor array processing. IEE Proc. Radar Sonar Navig..

[9] Paulraj A., Kailath T. Direction-of-arrival estimation by eigenstructure methods with unknown sensor gain-and-phase. Proceedings of the IEEE International Conference on Acoustics, Speech, and Signal Processing, ICASSP’85.

[10] Weiss A.J., Friedlander B. (1990). Eigenstructure methods for direction finding with sensor gain-and-phase uncertainties. Circuits Syst. Signal Process..

[11] Soon C., Tong L., Huang Y.F., Liu R. (1994). A subspace method for estimating sensor gains and phases. IEEE Trans. Signal Process..

[12] Wylie M.P., Roy S., Messer H. (1994). Joint DOA estimation and phase calibration of linear equispaced (LES) arrays. IEEE Trans. Signal Process..

[13] Li Y., Er M.H. (2006). Theoretical analyses of gain-and-phase error calibration with optimal implementation for linear equispaced array. IEEE Trans. Signal Process..

[14] Kim J., Yang H.J., Jung B.W., Chun J. (2010). Blind calibration for a linear array with gain-and-phase error using independent component analysis. IEEE Antennas Wirel. Propag. Lett..

[15] Zhao L., Liu H., Li Y., Zhou Y. DOA estimation under sensor gain-and-phase uncertainties. Proceedings of the International Conference on Estimation, Detection and Information Fusion.

[16] Liu A., Liao G., Zeng C., Yang Z., Xu Q. (2011). An eigenstructure method for estimating DOA and sensor gain-phase errors. IEEE Trans. Signal Process..

[17] Cao S., Ye Z., Xu D., Xu X. (2013). A Hadamard product based method for DOA estimation and gain-phase error calibration. IEEE Trans. Aerosp. Electron. Syst..

[18] Shechtman Y., Eldar Y.C., Cohen O., Chapman H.N., Miao J., Segev M. (2015). Phase retrieval with application to optical imaging: A contemporary overview. IEEE Signal Process. Mag..

[19] Jaganathan K., Oymak S., Hassibi B. (2017). Sparse phase retrieval: Uniqueness guarantees and recovery algorithms. IEEE Trans. Signal Process..

[20] Wang S., Zhang L., Jing X. (2017). Phase retrieval motivated nonlinear mimo communication with magnitude measurements. IEEE Trans. Wirel. Commun..

[21] Miao J., Ishikawa T., Shen Q., Earnest T. (2008). Extending X-ray crystallography to allow the imaging of noncrystalline materials, cells, and single protein complexes. Annu. Rev. Phys. Chem..

[22] Hüe F., Rodenburg J.M., Maiden A.M., Sweeney F., Midgley P.A. (2010). Wave-front phase retrieval in transmission electron microscopy via ptychography. Phys. Rev. B.

[23] Harrison R.W. (1993). Phase problem in crystallography. J. Opt. Soc. Am. A.

[24] Bunk O., Diaz A., Pfeiffer F., David C., Schmitt B., Satapathy D.K., van der Veen J.F. (2007). Diffractive imaging for periodic samples: Retrieving one-dimensional concentration profiles across microfluidic channels. Acta Crystallogr. Sect. A Found. Crystallogr..

[25] Fienup C., Dainty J. (1987). Phase retrieval and image reconstruction for astronomy. Image Recovery: Theory Application.

[26] Walther A. (1963). The question of phase retrieval in optics. Opt. Acta Int. J. Opt..

[27] Kim H., Haimovich A.M., Eldar Y.C. (2015). Non-coherent direction-of-arrival estimation from magnitude-only measurements. IEEE Signal Process. Lett..

[28] Qian C., Sidiropoulos N.D., Huang K., Huang L., Sidiropoulos N.D. (2016). Phase retrieval using feasible point pursuit: Algorithms and Cramér–Rao bound. IEEE Trans. Signal Process..

[29] Mehanna O., Huang K., Gopalakrishnan B., Konar A., Sidiropoulos N.D. (2015). Feasible point pursuit and successive approximation of non-convex QCQPs. IEEE Signal Process. Lett..

[30] Grant M., Boyd S., Ye Y. CVX: Matlab software for disciplined convex programming. http://cvxr.com/cvx/.

